# Association of Polymorphisms in X-Ray Repair Cross Complementing 1 Gene and Risk of Esophageal Squamous Cell Carcinoma in a Chinese Population

**DOI:** 10.1155/2015/509215

**Published:** 2015-02-01

**Authors:** Yu-Xia Yun, Li-Ping Dai, Peng Wang, Kai-Juan Wang, Jian-Ying Zhang, Wei Xie

**Affiliations:** ^1^Department of Radiology, The First Affiliated Hospital & Department of Epidemiology, College of Public Health and Henan Key Laboratory of Tumor Epidemiology, Zhengzhou University, Zhengzhou, Henan 450052, China; ^2^Department of Immunization Planning, Centers for Disease Control and Prevention of Puyang City, Puyang, Henan 457000, China

## Abstract

*Objectives*. To investigate the association between three single nucleotide polymorphisms (SNPs) in the X-ray repair cross complementing 1 gene (*XRCC1*) and the risk of esophageal squamous cell carcinoma (ESCC) in Chinese population. *Methods*. A case-control study including 381 primary ESCC patients recruited from hospital and 432 normal controls matched with patients by age and gender from Chinese Han population was conducted. The genotypes of three *XRCC1* polymorphisms at −77T>C (T-77C), codon 194 (Arg194Trp), and codon 399 (Arg399Gln) were studied by means of polymerase chain reaction-restriction fragment length polymorphism techniques (PCR-RFLP). Unconditional logistic regression model and haplotype analysis were used to estimate associations of these three SNPs in *XRCC1* gene with ESCC risk. *Results*. Polymorphisms at these three sites in *XRCC1* gene were not found to be associated with risk for developing ESCC; however the haplotype C_codon 194_G_codon 399_C_−77T>C_ was significantly associated with reduced risk of ESCC (OR: 0.62, 95% CI: 0.40–0.96) upon haplotype analysis. *Conclusion*. These results suggested that the gene-gene interactions might play vital roles in the progression on esophageal cancer in Chinese Han population and it would be necessary to confirm these findings in a large and multiethnic population.

## 1. Introduction

Esophageal cancer (EC) is the eighth most common malignancy and the sixth most common cause of cancer-related deaths worldwide, responsible for 3.8% of all new cancer cases and for 5.4% of cancer related deaths each year [1–5]. In 2008, about 482,000 new cases occurred and 406,000 patients died from EC worldwide, over 83% of which were in developing countries [2]. EC is the fourth leading cause of cancer death in China [6], with approximately 250,000 cases diagnosed yearly, and it contributes to about half of the cases of the world [4, 5, 7–10].

Base excision repair (BER) is one of the important DNA repair pathways against DNA damage that could lead to cancer resulted from many factors, including altered metabolism, reactive oxygen species, and methylating and deaminating agents [7–10]. The BER pathway has a primary role in the repair of oxidative base lesions such as 8-hydroxyguanine, formamidopyrimidines, and 5-hydroxyuracil [11]. Oxidative damage to DNA may lead to mutations that activate oncogenes or inactivate tumor suppressor genes and may eventually increase the probability of genetic alterations developing into neoplastic events [11]. Sequence variants in BER genes are thought to modulate DNA repair capacity and are consequently suspected of being associated with altered cancer risk [12]. XRCC1 protein encoded by* XRCC1* gene plays a critical role involved in the BER pathway, which interacts with enzymatic components of each stage of DNA strand break repair, including DNA polymerase beta, APE1 (apurinic/apyrimidinic endonuclease 1), PARP-1 (poly [ADP-ribose] polymerase 1), and DNA ligase III [13–18]. There are more than 60 validated single nucleotide polymorphisms (SNPs) in the* XRCC1* gene containing 17 exons and 16 introns on chromosome 19q13.2-13.3, among which three polymorphisms in the* XRCC1* gene at the −77T>C (5′ end, T to C), codon 194 (exon 6, Arg to Trp), and codon 399 (exon 10, Arg to Gln) have been studied.

The genetic polymorphism of* XRCC1* codon 194 results in an arginine to tryptophan amino acid substitution and occurs at a conserved residue in humans, hamsters, and mice, and this evolutionary conservation suggests that this site is functionally important [19, 20]. The genetic polymorphism in the* XRCC1* gene at codon 399 results in an arginine to glutamine amino acid substitution. A report of Lunn and colleagues measured the prevalence of aflatoxin B1 adducts in placental DNA from 120 Taiwanese women and suggested that the* XRCC1* codon 399 polymorphism may result in deficient DNA repair capacity [21]. The result of Hao et al. [22] showed that −77T>C can increase the combination of* XRCC1* promoter and a transcription inhibitory factor, thus reducing the promoter activity and protein expression. Because amino acid residues at the protein-protein interfaces of multiprotein complexes and residues involved in the active sites play a role in enzyme function, it is possible that* XRCC1* polymorphisms lead to alteration of DNA repair capacity.

Thus, we conducted this case-control study to comprehensively investigate the role of the polymorphisms (codon 194, codon 399, and −77T>C) in* XRCC1* gene in the development of ESCC in a Chinese Han population.

## 2. Materials and Methods

### 2.1. Study Population and Sample Collection

This case-control study included 381 newly histopathologically diagnosed primary ESCC patients recruited at the First Affiliated Hospital and the Second Affiliated Hospital of Zhengzhou University between March 2008 and January 2010. All ESCC patients had no prior history of other types of cancer and were not previously treated with chemotherapy or radiotherapy. Uniform trained investigators used the special questionnaire containing information of age, sex, tobacco smoking, alcohol intake, family history of cancer, and environmental factors to interview patients face to face after written informed consent. 432 normal controls were frequently matched by age (±5 years) and gender with patients randomly selected from a census of digestion diseases that we had conducted previously from March 2003 to July 2005 in Xinxiang County of Henan Province. Controls were required to be free of any digestion diseases with written informed consent, having no cancer history and related clinical signs. All the subjects were ethnic Han Chinese without immediate family relations.

The 5 mL venous blood samples obtained from the subjects were collected in an EDTA tube and stored at −70°C for extraction of DNA genome. Tobacco smoking was defined as smoking at least one cigarette per day and persisting for more than one year. Alcohol intake was defined as drinking at least once a week with more than 100 gram every time and persisting for more than six months. This study was approved by the Institutional Review Board of Zhengzhou University. Informed consent was obtained from each study participant.

### 2.2. *XRCC1* Genotyping

The genotypes of* XRCC1* polymorphisms were determined by PCR-RFLP. Primers and restriction endonucleases are shown in Table 1.

The PCR reaction started with a total volume of 15 *μ*L for each mixture containing the following reagents: 7.5 *μ*L 2 × Taq PCR MasterMix, 0.5 *μ*M each primer, 1.0 *μ*L (50 ng) DNA, and 5.6 *μ*L deionized water.

PCR conditions were as follows:

 codon 194: 95°C for 2 min, followed by 35 cycles of 94°C for 30 s, 60°C for 30 s, 72°C for 45 s, and a final elongation step at 72°C for 7 min,
 codon 399: 94°C for 5 min, followed by 35 cycles of 94°C for 30 s, 57°C for 30 s, 72°C for 45 s, and a final elongation step at 72°C for 5 min,
 −77T>C: 94°C for 5 min, followed by 35 cycles of 94°C for 30 s, 60°C for 30 s, 72°C for 30 s, and a final elongation step at 72°C for 5 min.

The digested products were resolved on 2% agarose gels (for codons 194 and 399), 3% agarose gels (for −77T>C) and stained with 0.5 *μ*g/mL ethidium bromide.

All assays were repeated at least once by the same individual it is not relevant to verify the genotyping results. Genotypes were validated by sequencing through biological technology company.

### 2.3. Statistical Analysis

All analyses were conducted by SPSS16.0 software. To determine whether the frequencies between cases and controls were significantly different (*α* = 0.05), the *χ* ^2^ test was used. And *χ* ^2^ test was also used to compare distribution differences of haplotype, combined genotype, the number of mutation sites, and the number of mutation alleles among three genetic polymorphisms.

Online software http://www.had2know.com/academics/hardy-weinberg-equilibrium-calculator-2-alleles.html was used to assess Hardy-Weinberg equilibrium for genotype frequency in controls by a Pearson's goodness-of-fit *χ* ^2^ test to compare the observed genotype frequencies to the expected ones, with one degree of freedom. Odds ratios (ORs) and 95% confidence intervals (95%CI) from logistic regression analysis for crude ORs and adjusted ORs when adjusting for age, gender, smoking, drinking, and family history of cancer were used to detect the associations between these three polymorphisms and ESCC risk. Haplotypes for each individual were inferred using the SNPHAP 2.0 software. Statistical tests were two sided and were considered statistically significant whenever *P* < 0.05.

## 3. Results

### 3.1. Subject Characteristics

The frequency distribution of age, sex, smoking, alcohol drinking status, and family history of cancer among the study subjects is summarized in Table 2. The number of individuals with a family history of cancer was higher in ESCC cases than in normal controls (*χ* ^2^ = 10.60, *P* = 0.001).

### 3.2. ESCC Risk Associated with Individual SNPs

The genotype distributions of the three studied SNPs in controls were all in accordance with Hardy-Weinberg equilibrium; the *P* value was 0.28, 0.79, and 0.09 for codon 194, codon 399, and −77T>C, respectively.

As shown in Table 3, the distributions of heterozygous and homozygous genotypes of* XRCC1* codon 194 and codon 399 did not show statistically significant difference between the cases and controls (*P* > 0.05), and the results were the same after adjustment for age, gender, smoking, drinking, and family history of cancer. The heterozygous genotype TC, combined genotype TC or CC, and allele C of* XRCC1* −77T>C may decrease risk of ESCC; however, there was no significantly statistical association after adjusting age, gender, smoking, drinking, and family history of cancer.

### 3.3. Haplotype Analysis

In order to study the haplotypes for* XRCC1* codon 194, codon 399, and −77T>C, SNPHAP 2.0 software was applied, showing a total of eight built haplotypes.  Table 4 shows the frequencies of the estimated haplotypes among patients and controls. For every susceptibility analysis of a haplotype, the haplotype CGT (codon 194, codon 399, and −77T>C) containing major allele was taken as control. The data of Table 4 indicated that only haplotype CGC (codon 194, codon 399, and −77T>C) may decrease risk for developing ESCC compared to the control after adjusting age, gender, smoking, drinking, and family history of cancer (OR: 0.62, 95% CI: 0.40–0.96).

### 3.4. Combined Genotypes Analysis of* XRCC1* Codon 194, Codon 399, and −77T>C

Using combined wild genotype CC/GG/TT as control, combined mutation genotype CT/GG/CT may decrease the risk in developing ESCC (OR: 0.46, 95% CI: 0.22–0.96, *P* = 0.04). But after adjusting age, gender, smoking, drinking, and family history of cancer, combined mutation genotype CT/GG/CT has no statistical association with ESCC (Table 5).

### 3.5. Trend Analysis of the Number of Mutation Sites

We performed trend analysis of the association between the number of mutation sites and risk of ESCC. Using null-mutation of three SNPs as control, there was no statistically significant association of one site mutation, two sites mutation, and three sites mutation with ESCC susceptibility (trend *χ* ^2^ = 0.38, *P* = 0.54) (Table 6).

## 4. Discussion

Environmental and genetic factors as identifiable risk factors are considered to have a significant contribution in the development of cancer. Some studies have found that environmental factors including tobacco smoking, alcohol consumption, nitrates, and preformed nitrosocompounds can increase EC risk [23, 24]. However, not all individuals who have been exposed to the environmental risk factors actually develop EC, suggesting that genetic susceptibility might contribute to the individual risk of EC.* XRCC1* polymorphisms have been reported to be associated with the risk of different kinds of cancers, including gastric cancer, colorectal cancer, lung cancer, and breast cancer [25–28].

Our data showed that there was no significant association between polymorphism of* XRCC1* codon 194 and ESCC susceptibility, and some results of the domestic and foreign researches showed to be consistent with ours. According to these results, Casson et al. [29] founded that there was no relationship between polymorphism of* XRCC1* codon 194 and esophageal adenocarcinoma (EA) susceptibility in Canada population. Lee et al.'s [30] study also found that there was no association between polymorphism of* XRCC1* codon 194 and ESCC in Asia population. On the contrary, there are studies showing that* XRCC1* codon 194 can increase the risk for EC. For example, the result of Xing et al. [31] indicated that the mutation homozygous genotype (TT) of* XRCC1* codon 194 is the risk factor for EC, the adjusted OR (95% CI) has a value of 1.98 (1.26–3.12). A case-control study of Liu et al. [32] in Hebei population of China found that the mutation homozygous genotype (TT) of* XRCC1* codon 194 can increase individual risk of ESCC 0.86-fold (adjusted OR (95% CI): 1.86 (1.19–2.88)).

Regarding the* XRCC1* codon 399 polymorphism results, we have not seen any significant association with ESCC. According to this, Chen et al. [33] and Song et al. [34] conducted a case-control study in Jiangsu and Henan provinces of China, respectively, showing no association between* XRCC1* codon 399 polymorphism and EC susceptibility. However, according to the results of Yu et al. [35], comparing with wild type (GG), the mutation homozygous genotype (AA) was a risk factor for developing EC (OR (95% CI): 5.15 (2.42–10.93)). By stratification analysis, smokers carrying homozygous mutant (AA) were associated with a 7.31-fold increased risk for EC compared to smokers carrying the wild type (GG) (OR: 8.31, 95% CI: 3.92–17.63), meanwhile drinkers carrying homozygous mutant (AA) were associated with a 4.43-fold increased risk for EC compared to drinkers carrying the wild type (GG) (OR: 5.43, 95% CI: 2.46–11.99).

In this study we did not observe significant association between polymorphism of* XRCC1* −77T>C and EC susceptibility. Supporting these results, as Hao et al. [22] firstly reported the* XRCC1* −77T>C polymorphism in 5′ noncoding region in 2004, the results found that* XRCC1* −77T>C can increase the combination of* XRCC1* promoter and a transcription inhibitory factor, thus reducing the promoter activity and protein expression. However, they did not find the association of* XRCC1* −77T>C polymorphism with ESCC susceptibility. The relationship between* XRCC1* −77T>C and EC susceptibility needs to be clarified by more large size studies.

In this study, we conducted quality control strictly throughout the whole study. The patients included were all newly pathologically diagnosed, thus avoiding the prevalence-incidence bias. Besides, the controls were frequency-matched and the investigators were unified-trained rigorously. Moreover, we sequenced the three SNPs duplicately and verified them by DNA sequencing making the results credible.

In conclusion, the present study suggested that the single polymorphism in the DNA repair gene* XRCC1 *was not statistically associated with risk of ESCC; however, haplotype CGC (codon 194, codon 399 and −77T>C) of three SNPs in* XRCC1* gene may decrease risk for ESCC susceptibility. But, in the future, it would be necessary to confirm these findings in a large and multiethnic population study because of the relatively small sample size in this study and gene-environment interaction analysis and it should also be performed to investigate the tumorigenesis mechanism.

## Figures and Tables

**Table 1:** Primer sequences and restriction endonucleases of three SNPs in *XRCC1* gene.

SNPs	Location	Position	Primers	Enzymes	Digested fragments
codon 194	exon 6	C26304T	F: GCCAGGGCCCCTCCTTCAA R: TACCCTCAGACCCACGAGT	PvuII	CC (485) CT (485, 396, 89) TT (396, 89)

codon 399	exon 10	G28152A	F: TTGTGCTTTCTCTGTGTCCA R: TCCTCCAGCCTTTTCTGATA	MspI	GG (374, 241) GA (615, 374, 241) AA (615)

−77T>C	5′UTR	T-77C	F: GGTTCTGGAAGCCACTCA R: GGGCTGAGGGCCTAAAC	BsrB I	TT (167, 67) TC (234, 167, 67) CC (234)

**Table 2:** Characteristics of esophageal cancer cases and controls.

Variables	Case *N* (%) *N* = 381	Control *N* (%) *N* = 432	*χ* ^2^	*P *
Age				
≤65	237 (62.2)	286 (66.2)	1.41	0.23
>65	144 (37.8)	146 (33.8)		
Gender				
Male	256 (67.2)	278 (64.4)	0.72	0.40
Female	125 (32.8)	154 (35.6)		
Smoking^*^				
Nonsmokers	189 (59.6)	264 (61.1)	0.17	0.68
Smokers	128 (40.4)	168 (38.9)		
Drinking^*^				
Nondrinkers	255 (80.7)	353 (81.9)	0.18	0.68
Drinkers	61 (19.3)	78 (18.1)		
Family history of cancer^*^				
No	233 (75.6)	357 (85.2)	10.60	0.001
Yes	75 (24.4)	62 (14.8)		

^*^because of the failure data collection, the number of cases and controls for some factors was less than 381 or 432.

**Table 3:** Association of *XRCC1* genotypes with esophageal cancer.

Genotypes	Case *N* (%)	Control *N* (%)	*χ* ^2^	OR (95% CI)	*P*	OR (95% CI)^*^	*P* ^*^
Codon 194							
CC	166 (46.6)	187 (44.6)	Reference	1.00	—	—	—
TC	159 (44.7)	193 (46.1)	0.24	0.93 (0.69, 1.25)	0.62	0.91 (0.66, 1.26)	0.57
TT	31 (8.7)	39 (9.3)	0.18	0.90 (0.53, 1.50)	0.67	0.96 (0.55, 1.68)	0.90
CC + TC	190 (53.4)	232 (55.4)	0.31	0.92 (0.69, 1.23)	0.58	0.92 (0.68, 1.25)	0.59
C	567 (67.7)	491 (69.0)	Reference	1.00	—	—	—
T	271 (32.3)	221 (31.0)	0.30	1.06 (0.86, 1.32)	0.58	0.90 (0.76, 1.21)	0.70
Codon 399							
GG	184 (48.5)	223 (51.6)	Reference	1.00	—	—	—
GA	166 (43.8)	169 (40.7)	1.39	1.19 (0.89, 1.59)	0.24	1.14 (0.84, 1.57)	0.40
AA	29 (7.7)	30 (7.7)	0.32	1.17 (0.68, 2.02)	0.57	1.03 (0.57, 1.86)	0.92
GA + AA	195 (51.5)	199 (47.2)	1.47	1.47 (0.22, 1.57)	0.22	1.13 (0.83, 1.52)	0.44
G	534 (70.4)	615 (72.9)	Reference	1.00	—	—	—
A	224 (29.6)	229 (27.1)	1.15	1.13 (0.91, 1.40)	0.28	1.07 (0.85, 1.35)	0.57
−77T>C							
TT	310 (83.6)	319 (77.2)	Reference	1.00	—	—	—
TC	59 (15.9)	92 (22.3)	5.09	0.66 (0.46, 0.95)	0.02	0.70 (0.48, 1.03)	0.07
CC	2 (0.5)	2 (0.5)	0.22	1.03 (0.14, 7.35)	0.64	0	1.0
TC + CC	61 (16.4)	94 (22.8)	4.92	0.67 (0.47, 0.96)	0.03	0.69 (0.47, 1.01)	0.06
T	679 (91.5)	730 (88.4)	Reference	1.00	—	—	—
C	63 (8.5)	96 (11.6)	4.37	0.70 (0.50, 0.98)	0.04	0.70 (0.48, 1.00)	0.05

^*^adjusted for age, gender, smoking, drinking, and family history of cancer.

**Table 4:** * XRCC1* haplotype analysis of three polymorphisms in *XRCC1 * gene.

Haplotypes	Case *N* (%)	Control *N* (%)	*χ* ^2^	OR (95% CI)	*P *	OR (95% CI)^*^	*P* ^*^
CGT	323 (42.4)	349 (40.4)	Reference	1.00	—	—	—
TGT	152 (19.9)	189 (21.9)	1.11	0.87 (0.67, 1.13)	0.29	0.81 (0.60, 1.10)	0.18
CAT	221 (29.0)	229 (26.5)	0.12	1.04 (0.82, 1.32)	0.73	0.99 (0.74, 1.31)	0.92
CGC	57 (7.5)	89 (10.3)	3.93	0.69 (0.48, 1.00)	0.05	0.62 (0.40, 0.96)	0.03
CAC	2 (0.3)	4 (0.5)	0.10	0.54 (0.10, 2.97)	0.76	0.25 (0.03, 2.30)	0.22
TAT	3 (0.4)	1 (0.1)	0.33	3.24 (0.34, 31.32)	0.57	3.60 (0.36, 35.79)	0.27
TGC	4 (0.5)	3 (0.3)	0.01	1.44 (0.32, 6.49)	0.92	0.76 (0.10, 5.72)	0.79
TAC	0	—	—	—	—	—	—

Total	762 (100)	864 (100)	—	—	—	—	—

^*^adjusted for age, gender, smoking, drinking, and family history of cancer.

The order of three SNPs: codon 194, codon 399, and −77T>C.

**Table 5:** Combination genotypes analysis of *XRCC1* codons 194, 399, and −77T>C.

Combined genotypes	Case *N* (%)	Control *N* (%)	*χ* ^2^	OR (95% CI)	*P*	OR (95% CI)^*^	*P* ^*^
CC/GG/TT	39 (11.2)	43 (10.6)	Reference	1.00	—	—	—
CT/GA/TT	68 (19.5)	71 (17.6)	0.04	1.06 (0.61, 1.82)	0.85	0.95 (0.53, 1.72)	0.87
CT/GG/TT	65 (18.7)	72 (17.8)	0.00	0.99 (0.58, 1.72)	0.99	0.88 (0.49, 1.60)	0.68
CC/GA/TT	60 (17.2)	66 (16.3)	0.00	1.00 (0.57, 1.75)	0.99	1.02 (0.56, 1.87)	0.95
TT/GG/TT	29 (8.3)	37 (9.2)	0.19	0.86 (0.45, 1.66)	0.66	0.91 (0.45, 1.84)	0.80
CT/GG/CT	16 (4.6)	38 (9.4)	4.35	0.46 (0.22, 0.96)	0.04	0.52 (0.24, 1.13)	0.10
CC/AA/TT	23 (6.6)	23 (5.7)	0.07	1.10 (0.53, 2.27)	0.79	0.86 (0.39, 1.92)	0.72
CC/GA/CT	17 (4.9)	23 (5.7)	0.28	0.81 (0.38, 1.75)	0.60	0.74 (0.32, 1.70)	0.48
CC/GG/CT	20 (5.7)	22 (5.4)	0.00	1.00 (0.48, 2.11)	1.00	1.09 (0.50, 2.42)	0.82
CT/GA/CT	4 (1.1)	2 (0.5)	0.23	2.21 (0.38, 12.71)	0.63	1.18 (0.18, 7.74)	0.86
CC/AA/CT	1 (0.3)	3 (0.7)	0.14	0.37 (0.04, 3.68)	0.71	0.37 (0.04, 3.87)	0.41
CT/AA/TT	3 (0.9)	1 (0.2)	0.31	3.31 (0.33, 33.13)	0.58	3.74 (0.36, 38.83)	0.27
Others	3 (0.9)	3 (0.9)	—	—	—	—	—
Total	**348 (100)**	**404 (100)**	**—**	**—**	**—**	**—**	**—**

^*^adjusted for age, gender, smoking, drinking, and family history of cancer.

**Table 6:** Trend analysis of the number of mutation sites.

Number of mutation sites	Case *N* (%)	Control *N* (%)	*χ* ^2^	OR (95% CI)	*P*	OR (95% CI)^1^	*P* ^1^
0	39 (11.2)	43 (10.6)	Reference	1.00	—	—	—
1	198 (56.9)	220 (54.5)	0.00	0.99 (0.62, 1.59)	0.97	0.95 (0.57, 1.59)	0.84
2	107 (30.7)	139 (34.4)	0.41	0.85 (0.51, 1.40)	0.52	0.78 (0.46, 1.36)	0.40
3	4 (1.1)	2 (0.5)	0.23	2.21 (0.38, 12.71)	0.63	1.23 (0.19, 8.04)	0.83
—	—	—	0.38^*^	—	0.54^*^	—	—

Total	348 (100)	404 (100)	—	—	—	—	—

^*^trend chi-square.

^1^Adjusted for age, gender, smoking, drinking, and family history of cancer.
